# Dexmedetomidine Improves Cardiovascular and Ventilatory Outcomes in Critically Ill Patients: Basic and Clinical Approaches

**DOI:** 10.3389/fphar.2019.01641

**Published:** 2020-02-28

**Authors:** Rodrigo L. Castillo, Mauricio Ibacache, Ignacio Cortínez, Catalina Carrasco-Pozo, Jorge G. Farías, Rodrigo A. Carrasco, Patricio Vargas-Errázuriz, Daniel Ramos, Rafael Benavente, Daniela Henríquez Torres, Aníbal Méndez

**Affiliations:** ^1^ Departamento de Medicina Interna Oriente, Facultad de Medicina, Universidad de Chile, Santiago, Chile; ^2^ Unidad de Paciente Crítico, Hospital del Salvador, Santiago, Chile; ^3^ Programa de Farmacología y Toxicología & División de Anestesiología, Facultad de Medicina, Pontificia Universidad Católica de Chile, Santiago, Chile; ^4^ Discovery Biology, Griffith Institute for Drug Discovery, Griffith University, Nathan, QLD, Australia; ^5^ Departmento de Ingeniería Química, Facultad de Ingeniería y Ciencias, Universidad de La Frontera, Francisco Salazar, Chile; ^6^ Departamento de Cardiología, Clínica Alemana-Universidad del Desarrollo, Santiago, Chile; ^7^ Unidad de Paciente Crítico Adulto, Clínica Universidad de Los Andes, Santiago, Chile; ^8^ Unidad de Paciente Crítico, Clínica Alemana-Universidad del Desarrollo, Santiago, Chile

**Keywords:** dexmedetomidine, cardiac, preconditioning, pharmacokinetics, non-invasive mechanical ventilation, sedative and analgesic properties

## Abstract

Dexmedetomidine (DEX) is a highly selective α2-adrenergic agonist with sedative and analgesic properties, with minimal respiratory effects. It is used as a sedative in the intensive care unit and the operating room. The opioid-sparing effect and the absence of respiratory effects make dexmedetomidine an attractive adjuvant drug for anesthesia in obese patients who are at an increased risk for postoperative respiratory complications. The pharmacodynamic effects on the cardiovascular system are known; however the mechanisms that induce cardioprotection are still under study. Regarding the pharmacokinetics properties, this drug is extensively metabolized in the liver by the uridine diphosphate glucuronosyltransferases. It has a relatively high hepatic extraction ratio, and therefore, its metabolism is dependent on liver blood flow. This review shows, from a basic clinical approach, the evidence supporting the use of dexmedetomidine in different settings, from its use in animal models of ischemia-reperfusion, and cardioprotective signaling pathways. In addition, pharmacokinetics and pharmacodynamics studies in obese subjects and the management of patients subjected to mechanical ventilation are described. Moreover, the clinical efficacy of delirium incidence in patients with indication of non-invasive ventilation is shown. Finally, the available evidence from DEX is described by a group of Chilean pharmacologists and clinicians who have worked for more than 10 years on DEX.

## Introduction

Dexmedetomidine (DEX), a pharmacological alpha 2-adrenergic agonist, which induces sympatholytic mechanism on the brain with anxiolytic and sedative effects (Cormack et al., 2005). DEX triggers the sedative pathways, causing a type of mild sedation and minimizing the risk of respiratory depression. This drug is mainly used in two settings: (1) in intensive care for moderate sedation, although it is not recommended for long-term sedation; and (2) in anesthesia as a stand-alone sedative or as co-adjuvant for general anesthesia. In both settings, DEX has shown interesting cardiovascular, respiratory, anti-inflammatory, and organ protective properties. In clinic, it is used in perioperative sedation and intensive care, due to its analgesic and anxiolytics effects. The drug is commonly administered intravenously, either as a single bolus of approximately 1 μg/kg or as a bolus plus a continuous infusion ranging between 0.2 and 1 μg/kg/h. The stimulus of the different subtypes of α2-adrenergic receptors (α2A, α2B, α2C) and its location in the nervous system (pre or postsynaptic), will determine the different effects observed during use. Currently, many biological functions of DEX have been demonstrated in different animal models, through several signaling pathways.

Currently, the biological functions of DEX have been demonstrated in different animal models through several signaling pathways. Some studies demonstrate the beneficial effects of DEX against ischemia–reperfusi0on (I/R) injury (Riquelme et al., 2016; Deng et al., 2019), activating the eNOS/NO cardioprotective signaling pathways. *In vivo* mechanistic studies are needed to determine the effects of DEX in clinical events that are associated with I/R. This review shows that DEX may be a pharmacological agent that modulates the organ I/R injury responses in humans.

## Pharmacokinetic Properties

DEX is an imidazole derivative with a 236.7 g/mol molecular mass and a 2.89 octanol/water partition coefficient (Reel, 2019). Loading doses and infusion rates are determined on a milligram per kilogram total body weight (TBW). In general, linear pharmacokinetics adequately describes the body disposition of the drug, even after prolonged high dose administration in critically ill patients (Iirola et al., 2011a; Välitalo et al., 2013). However, patients with severe hepatic failure or obstructive jaundice have shown a reduction of metabolic clearance (CL) and significant changes in the volume of distribution (VD) (Cunningham et al., 1999; Song et al., 2018). DEX CL remains stable with dose increments within the therapeutic range and decreases with the administration of supratherapeutic doses (Dutta et al., 2000; Iirola et al., 2012).

Even though DEX was developed for intravenous use, it has been administered by different routes with variable bioavailability. Intramuscular administration has shown bioavailability of 103.6% with a time to peak of 1.7 ± 1.8 h (Anttila et al., 2003). Nasal or buccal (submucosal) administration has been successfully used in patients without available venous access, avoiding high plasma peak levels. The bioavailability and time to peak of the nasal approach is 65% (35–93%) and 38 (15–60) min, respectively (Iirola et al., 2011b; Yoo et al., 2015). The bioavailability and time to peak of the buccal route is 81.8% (72.6–92.1%) and 1.5 ± 0.2 h, respectively. Due to an extensive first-pass effect, the bioavailability of the drug reaches only 15.6% after oral administration (Anttila et al., 2003). DEX has a high protein binding (94%) with an extensive VD and easily crosses the blood-brain barrier (Bhana et al., 2000). In non-compartmental kinetics, the administration of a single bolus has a 6.5 ± 3.4 min distribution half-life (Anttila et al., 2003). The drug has a described steady state VD of 80–194 l, which is related to patient weight (Dyck et al., 1993; Khan et al., 1999; Välitalo et al., 2013). ICU patients tend to have greater variability of this parameter (109–223 l), and hypoalbuminemia has been shown to increase the VD in these patients (Iirola et al., 2012; Hodiamont et al., 2017).

The drug is extensively metabolized in the liver with a determined extraction ratio of 0.7, and less than 1% of the drug eliminated without changes (Anttila et al., 2003). DEX undergoes N-glucuronidation (34%) by uridine diphosphate glucuronosyltransferases (UGT2B10, UGT1A4) and is also hydroxylated in a smaller proportion by the P450 enzyme system, specifically CYP2A6 (Adams and Murphy, 2000; Jorden and Tung, 2002; Kohli et al., 2012). The generated metabolites are approximately 100 times less potent than the original administered drug and are considered inactive. These metabolites are finally eliminated *via* the kidneys (95%). The CL is 36–42 l/h in average adult patients (Dyck et al., 1993; Khan et al., 1999). In ICU patients, CL has been defined at 31.8–57 l/h (Venn et al., 2002; Zhang et al., 2015). In healthy volunteers, the elimination half-life is 2.1–3.1 h, and in ICU patients, the half-life slightly increases to 2.2–3.7 h (Karol and Maze, 2000; Venn et al., 2002; Zhang et al., 2015). In this context, the presence of hypoalbuminemia decreases or increases half-life times (Iirola et al., 2011a; Zhang et al., 2015). However, as a drug with a high hepatic extraction ratio, DEX CL would depend more on the blood flow to the liver (cardiac output) than on protein binding (Benet, 2002). It has been reported that a 19% cardiac output reduction decreases DEX CL by 12% (Dutta et al., 2000).

Numerous factors influencing the DEX dosage have been reported in the literature. Conditions such as hypoalbuminemia, liver dysfunction, reduced cardiac output, and hemodynamic alterations significantly affect both the VD and CL (Karol and Maze, 2000; Dutta et al., 2000; Zhang et al., 2015). Several pharmacokinetic models developed in different populations show that observed inter-individual variability of DEX body disposition is explained by the influence of the aforementioned covariates on pharmacokinetic parameters and patient dose requirements. Other covariates, such as ethnicity and polymorphisms of metabolic enzymes, are less relevant contributors to the described variability (Karol, 1996; Karol and Maze, 2000; Kurnik et al., 2008; Kohli et al., 2012).

In adults, the majority of these experimental models were derived from four trials of critically ill patients or healthy volunteers in which different DEX continuous infusions with varying durations were used. In most studies, a 2-compartment model adequately describes the disposition and elimination of DEX (Khan et al., 1999; Venn et al., 2002; Zhang et al., 2015; Cortinez et al., 2015). Fewer trials have described models of 1 and 3 compartments (Dyck et al., 1993; Dutta et al., 2000; Lin et al., 2011; Hannivoort et al., 2015). In these models, the VD has been correlated with patient age, TBW, free-fat mass (FFM), albuminemia, and the intercurrence of surgery. The CL varies according to patient height, TBW, FFM, albuminemia, and alanine aminotransferase metabolic activity. The review article by Weerink et al. evaluated the impact of different covariates on the DEX plasma concentration-time profile by means of comprehensive simulations (Weerink et al., 2017). For this purpose, a DEX 35 µg loading dose infused over 10 min, followed by a 35 µg/h maintenance dose was simulated supporting the different published pharmacokinetic simulations. In a 2h DEX simulated infusion the authors found: 1) DEX reached concentrations after a bolus dose was directly dependent on patient albuminemia and inversely related to patient height, with a less significant influence of age on the observed maximum concentration; 2) the accomplished concentration during DEX infusion (steady state) was influenced by patient age, albuminemia, height, and body weight. These findings are in agreement with previously published data where age-associated metabolic CL reduction increases DEX plasma concentration in patients (Iirola et al., 2012). Albuminemia and height may also influence DEX concentrations, but their impact is less clear compared to other more relevant covariates (Lin et al., 2011; Lee et al., 2012; Zhang et al., 2015). For instance, available evidence suggests that DEX CL is significantly influenced by hepatic blood flow and cardiac output, but since DEX likely has a high hepatic extraction ratio, this influence is difficult to establish in all populations. However, the non-linear behavior of DEX CL has been described in non-compartmental and compartmental analyses where DEX concentration increments reduce the drug CL by reducing cardiac output (Dutta et al., 2000; Iirola et al., 2012). At present, changes in TBW or FFM have a significant impact on concentrations after a bolus and during the infusion of DEX and should be considered when dosing adult patients (Cortinez et al., 2015; Hannivoort et al., 2015; Kuang et al., 2016).

As previously mentioned, TBW emerges as a significant covariate that influences the concentrations of DEX. Infusion schemes using mass units of drug per kilogram of TBW do not seem appropriate for the obese individual, as they result in increased plasma levels than those observed in lean subjects. In a pharmacokinetic modeling analysis by Cortinez et al., lean tissues showed as fat-free mass (FFM) accounted for size-dependent changes in VD of DEX (Cortinez et al., 2015). In addition, they also showed that for any lean body mass, total CL decreased associated with major fat mass. In a second trial by the same study group, authors confirmed that lean body mass was an appropriate dosing scalar for size in obese patients and showed that decreased hepatic blood had a signiﬁcant effect on the CL of DEX (Rolle et al., 2018). The observed CL reduction in this population is most probably explained by a relative overdose of obese patients caused by TBW-based dosing schemes. In this setting, scaling doses to FFM or allometric TBW seems more relevant (Cortinez et al., 2015; Hannivoort et al., 2015; Rolle et al., 2018).

Although the drug is not approved for clinical administration in the pediatric population, its off-label use is widely extended in these patients (Mahmoud and Mason, 2015). DEX pharmacokinetics in children has been studied in ICU patients and in patients undergoing cardiac and non-cardiac surgery (Potts et al., 2009; Su et al., 2010; Wiczling et al., 2016; Su F. et al., 2016; Liu et al., 2017). A 2-compartment model, using TBW scaled to 1 for the VD and 0.75 for the CL (Equation 1), adequately describes the disposition of the drug in pediatric populations.

(1) y=a〖TBW〗∧PWR

where y is the variable of interest (e.g., Cl or V), a is the allometric coefficient, and PWR is the allometric exponent.

In children, 93% of DEX is protein bound with a rapid redistribution half-life of approximately 7 min and elimination half-life of 2 h (Petroz et al., 2006; Mason and Lerman, 2011). Weight appears as a significant covariate influencing DEX CL in children older than 2 years with nearby levels (within 30–40%) to those reported in adults (Petroz et al., 2006). Several studies report the influence of the maturation of neonatal enzymatic activity on CL, but the available data are very dissimilar. At birth, a full-term neonate has a DEX CL of 43–54% adult values, and in a variable period (1 month–1 year) reaches adult values (Potts et al., 2009; Wiczling et al., 2016). Overall, allometric scaling can be used to predict DEX concentrations in children older than 1 year of age. In children younger than 1 year, inter-individual variability significantly influences the CL and is substantially greater than the impact of maturation on this parameter. Thus, despite considering maturation and patient age, it is difficult to achieve a certain predicted concentration in very small children.

## Pharmacodynamic Properties

In terms of the pharmacodynamic properties of DEX, it shows a high affinity and selectivity for the α2-adrenoceptors, this pharmacological characteristic determines a typical biphasic hemodynamic response. After infusion, DEX induces sympatholytic effect, such as a lower mean arterial blood pressure (MAP) and heart rate (HR) through activation of presynaptic α2-adrenoceptors in the central nervous system. In addition, DEX induces vasodilation through activation of α2-adrenoceptors in endothelial cells (Figueroa et al., 2001; Talke et al., 2003). At higher concentrations, vasoconstrictive effects of DEX are attributable to activation of α2-adrenoceptors in vascular smooth muscle, resulting clinically in an increase in MAP and diminution of HR (Snapir et al., 2006). The pharmacokinetic and pharmacodynamic effects of DEX have been extensively studied in animal models, however its role in critical patients’ arterial pressure and heart rate has been scarcely addressed (Ebert et al., 2000; Snapir et al., 2006; Potts et al., 2010).

### Adverse Effects

Adverse effects of dexmedetomidine are mainly restricted to hemodynamic alterations. These include hypertension, bradycardia, and hypotension owing to pre- and postsynaptic α2-receptor activation, which causes vasoconstriction, vasodilatation, and reflex bradycardia (Ebert et al., 2000). Long-term use of dexmedetomidine leads to super sensitization and upregulation of receptors. With abrupt discontinuation, a withdrawal syndrome of nervousness, agitation, headaches, and hypertensive crisis can occur.

Common adverse effects for organ systems (1 to 10%) are: respiratory, Atelectasis, hypoxia, pulmonary edema, pleural effusion, respiratory failure, acute respiratory distress syndrome, bradypnea, pneumonia, pharyngolaryngeal pain, gastrointestinal, dry mouth, vomiting, constipation, abdominal distension, abdominal pain, diarrhea, hematologic, anemia, metabolic, hyperglycemia, hypoglycemia, hypocalcemia, acidosis, hypokalemia, hypomagnesemia, hypernatremia, hypophosphatemia, acute renal failure, and oliguria (Cheung et al., 2014).

Dexmedetomidine is not recommended in patients with advanced heart block and ventricular dysfunction. FDA has classified it as a category C pregnancy risk, therefore the drug should be used with extreme caution in women who are pregnant (Morgan et al., 2006).

Two major limitations regarding dexmedetomidine use, are its long-lasting effects and its hemodynamic side effects. A safe and quick reversal of these effects would benefit clinical practice, presumably leading to more widespread use of dexmedetomidine. The selective α2-antagonist atipamezole can effectively reverse dexmedetomidine’s hemodynamic and sedative effects (Karhuvaara et al., 1991). The reduction in heart rate and blood pressure caused by dexmedetomidine is quickly reversed after IV administration of 15–150 µg/kg atipamezole. Higher doses of atipamezole (150 µg/kg) also reverse sedation. Transient orthosympathic activation, with a 10-fold increase in plasma norepinephrine levels, is seen with higher doses or fast infusion rates (Scheinin et al., 1998).

### Effects on Cardiovascular and Ventilatory Physiology

The US Food and Drug Administration (FDA) originally approved DEX for use in “initially intubated, mechanically ventilated patients,” that is, in ventilated patients requiring sedation throughout and after extubation. Then Hanna and Milap, (2008) approved its use for the sedation of non-invasive intubated critical patients (Panzer et al., 2009; Keating et al., 2012). Since DEX’s appearance on the market as a sedative drug, cardiovascular and ventilatory effects have been extensively characterized (Deutsch and Tobias, 2007).

The effects of DEX on the cardiovascular system are a consequence of the pharmacological modulation of the α-2 adrenergic receptor. The stimulation of the different subtypes of α2 adrenergic receptors (α2A, α2B α2C) and their location in the nervous system (pre or postsynaptic), will determine the different cardiovascular effects observed during its use. An intravenous bolus of DEX in healthy individuals determines a biphasic blood pressure response. DEX infusion induces an initial transient increase vasoconstriction in vascular smooth musculature (by activation of postsynaptic α2B receptors), followed by a decrease in blood pressure and heart rate (by the activation of α2A receptors in the central nervous system). This apparent dual action of the drug becomes evident when its effect is considered in sympathetically denervated territories, such as when an anesthetic blockade of peripheral nerve exists (Talke et al., 2003) or when high doses of the drug are used. In these situations, the peripheral vasoconstrictor effect of the drug predominates. The opposite effect, vasodilation associated with central sympatholysis is observed when low doses are used in subjects with a non-intervened sympathetic system. The omission of the DEX loading bolus prevents initial hypertension and reflex bradycardia (Ickeringill et al., 2004). In addition, stimulation of postsynaptic α2-adrenergic receptors in endothelial cells would also induce vasodilation. In this context, the correct infusion rate of the drug, appropriate volume administration and clinical indication, make DEX a substance with a wide security therapeutic range (Kamibayashi and Maze, 2000). The central inhibition of the sympathetic system could minimize patient stress and instability in blood pressure during and after cardiac surgery (Gong et al., 2017). Evidence suggests that induced autonomic nervous system modulation during perioperative administration of DEX is associated with a trend towards improved cardiac outcomes following noncardiac surgery (Biccard et al., 2008; Wijeysundera et al., 2009). In addition, this central inhibition of sympathetic discharge could prevent the sympathetic reservoir from being depleted, and therefore the Dex group has higher blood pressure compared to the control group. Mukhtar et al. used a dosage of 0.5 μg/kg/h by infusion, which was effective in diminishing the hemodynamic response to surgery without deleterious vasodepressor effects (Mukhtar, 2006).

Despite the deep sedative effects, DEX is associated with minor respiratory depression, even when it is dosed in plasma levels up to 15 times with those usually obtained during administration, offering a higher level of safety, compared with other sedative agents (Venn et al., 2000; Filbey et al., 2014). Hypercapnic stimulation is preserved and limits apnea or attenuates the stimulatory effect of CO2 levels. In volunteers, DEX infusions preserved the hypercapnic ventilatory response, the respiratory rate was significantly increased, and the overall apnea/hypopnea index was significantly decreased. The distribution of inspiratory time/ventilatory cycle time showed an increased peak. More importantly, DEX exhibited a hypercapnic arousal phenomenon similar to that which has been described during natural sleep (Hsu et al., 2004). Compared with opioid infusions, DEX can be infused by a tracheal tube in a safe manner (Panzer et al., 2009).

### DEX Molecular Pathways

DEX modulates some intracellular pathways such as G protein-coupled receptors, protein kinase C (PKC) activity, and inositol triphosphate (IP3) levels in renal cells. Protein kinase C is relevant as an ischemic preconditioning trigger providing organ protection, the opening mitochondrial ATP-sensitive K+ channels, and the induction of protective gene transcription (Curtis et al., 2011). DEX determines the *in vitro* activation of the adenylyl cyclase-cyclic adenosine monophosphate (cAMP), suggesting that DEX may have a protective effect through the modulation of PKC activation-induced HSP27 phosphorylation (Tanabe et al., 2008; Tanabe et al., 2010). The α2-AR-focal adhesion kinase-Src-phosphatidylinositol 3-kinase (PI3K)-protein kinase B (Akt) (Girault et al., 1999; Parcellier et al., 2008), and the mitochondrial ATP-sensitive K+ channel pathways are involved in the preconditioning and postconditioning mechanisms induced by DEX against hippocampal oxidative damage (Dahmani et al., 2008; Dahmani et al., 2010). Treatment with DEX reduces central nervous injury in rats subjected to focal I/R, which is mediated by the activation of the PI3K/Akt and ERK1/2 pathways (Zhu et al., 2013). Moreover, DEX attenuates hippocampal CA1 long-term potentiation. α2-ARs and imidazoline I2 receptors are modulated by DEX (Takamatsu et al., 2008). Regarding the neuroprotective effects, epidermal growth factor receptor activation in astrocytes *in vivo* represents an important process secondary to α2-AR activation (Du et al., 2009). Moreover, the α2-ARs also activate the mitogen-activated protein kinase pathway and thus determine the proliferation of tubular cells derived from the human intestinal epithelium in rats. (Schaak et al., 2000; Cussac et al., 2002; Karkoulias et al., 2006).

Previous animal experiments have shown benefits and cardioprotective effects of DEX administration in the ischemic heart (Roekaerts et al., 1996; Willigers et al., 2003; Okada et al., 2007). Furthermore, it has been shown that DEX administration causes activation of signaling pathways associated with cardiac survival. The DEX pretreatment of rat hearts induces Erk 1/2, Akt and eNOS activation, improves myocardial function and reduces myocardial infarction size after regional ischemia-reperfusion (I/R) of the heart in the *in vivo* and *ex vivo* models (Ibacache et al., 2012). In this study, the three isotypes of α2-adrenergic receptors were detected in the whole cardiac tissue and the α2A and α2C adrenergic receptors were detected only in isolated cardiomyocytes. The authors concluded that independently from autonomic nervous system modulation, DEX cardioprotective effects are mediated by activation of pro-survival PI3K/Akt signaling pathway after cardiac α2-adrenergic receptor stimulation. More recent studies have demonstrated the role of endothelial α2-adrenergic receptors with beneficial effects on DEX against I/R injury (Riquelme et al., 2016; Deng et al., 2019), activating the eNOS/NO cardioprotective signaling pathways (Kim et al., 2009).

### Molecular Mechanisms of Dexmedetomidine Effects on Heart and Lung

The mitochondria are critical coordinators of cellular life through their function on bioenergetics, producing energy, and their role in apoptosis, regulating cell death (Wang and Youle, 2009). The mitochondria supply 90% of the cardiomyocyte ATP, which is necessary to support contraction, metabolism, and ion homeostasis, and thus have a critical role in cardiac function (Harris and Das, 1991; Pohjoismäki and Goffart, 2017). Mitochondrial dysfunction has been involved in the pathophysiology of cardiovascular disease, including ischemia/reperfusion (I/R) injury, arrhythmogenesis, and left ventricular dysfunction (Lesnefsky et al., 2001; Chen et al., 2018a; Chen et al., 2018b). It has been shown that DEX preconditioning rat cardiomyocytes through mitochondrial protection diminishes reactive oxygen species (ROS)-induced apoptosis (Liu et al., 2018; Weng et al., 2018). Dexmedetomidine prevented the increase in the oxygen consumption rate and ROS levels induced by H2O2. It improved the coupling efficiency of the mitochondria potentially by preserving mitochondrial membrane potential (MMP) in the presence of mitochondrial uncouplers (Liu et al., 2018; Weng et al., 2018). Moreover, dexmedetomidine decreased the H2O2-induced activity of caspase 12, and the mRNA levels of Grp78 (glucose regulated protein 78 kDa) and IRE1α (serine/threonine protein kinase/endoribonuclease), three signaling pathways that determine ER stress mediated cell death (Liu et al., 2018). The cardioprotective effect of DEX has also been evidenced in isolated rat hearts, in which DEX preconditioning (10 nmol/l before ischemia for 15 min) had protective effects against I/R injury. The agonist attenuates myocardial cell death and improves cardiac function through mechanisms that involve the inhibition of the mitochondrial permeability transition pore (mPTP) opening at reperfusion time (Jiang et al., 2013). The opening of the mPTP results in respiratory chain uncoupling, mitochondrial ATP synthesis reduction, mitochondrial swelling and cell death (Hunter and Haworth, 1979; Halestrap, 2009). Thus, the mPTP is a selective pharmacological target for cell death prevention in pathophysiological conditions. Cyclophilin D, a regulator of the mPTP, protects against cell death in response to different injuries (Millay et al., 2008; Martin et al., 2009; Ramachandran et al., 2011). The protection of the mitochondrial function, by inhibiting the mPTP opening and thus preserving the MMP, is a plausible mechanism by which DEX exerts its cardioprotective effects.

As an anesthesia co-adjuvant, dexmedetomidine can improve hemodynamic stability and reduce the doses of anesthetics and analgesics during surgery, and it may contribute to the prevention of postoperative cognitive dysfunction (POCD) (Carr et al., 2018). In patients undergoing elective surgery under general anesthesia induced by remifentanil (0.5–1 µg/kg/h) and propofol (3–10 mg/kg/h), the addition of dexmedetomidine (0.2 µg/kg/h) during induction and maintenance periods improved POCD, through the mechanism involving mitochondrial function protection. Dexmedetomidine attenuated the decrease in postoperative National Institutes of Health Stroke Scale (NIHSS) and Auditory Verbal Learning Test (AVLT) scores and the increase in the Beck Depression Inventory (BDI) score. In terms of mitochondrial function, dexmedetomidine alleviated the postoperative decrease in mitochondrial membrane potentials and the activities of the mitochondrial respiratory complexes I–IV in leukocyte cells (Chen et al., 2018a; Chen et al., 2018b). Moreover, dexmedetomidine has been shown to have neuroprotective effects against apoptosis through mechanisms involving mitochondrial functions (Flatters and Bennett, 2006). Apoptosis is a major pathway in I/R‐induced neuronal and cardiac insults (Borgens and Liu-Synder, 2012). Dexmedetomidine prevented apoptosis induced by I/R by protecting against MMP reduction, Bax and cytochrome c release as well as caspase activation in neuro-2a and NB41A3 neuronal cells (Wu et al., 2017). Dexmedetomidine also protected PC12 cells from lidocaine-induced apoptosis, diminished the mRNA levels of COL3A1, increasing Bcl2, and inhibiting caspase 3 activation (Wang et al., 2017). Interestingly, this effect was reversed by the miR-let-7b inhibitor, indicating that dexmedetomidine exerts neuroprotection *via* miR-let-7b, a recognized modulator of mitochondrial function (Barrey et al., 2011; Wang et al., 2017). Dexmedetomidine protected PC12 cells against glutamate-induced cytotoxicity through its mitochondrial protective effect, stabilizing MMP and Ca2+ homeostasis, and through its antioxidant properties, reducing malondialdehyde and augmenting superoxide dismutase activity (Zhang et al., 2016).

In addition, DEX has demonstrated neuroprotective effects against ischemia or tissular hypoxia. In an *in vivo* neuroprotection model, DEX prevented the brain damage induced by cerebral ischemia-reperfusion in rats by activating mitochondrial ATP-sensitive potassium channel (mitoKATP) (Yuan et al., 2017). Dexmedetomidine (50 μg/kg injected intraperitoneally before ischemia and after onset of reperfusion) prevented the I/R-induced increase in the neurological deficit score, pro-oxidant enzyme activity (such as myeloperoxidase and malondialdehyde) in the brain and pro-inflammatory cytokine levels in plasma (like IL-6 and TNF-α) (Yuan et al., 2017). The neuroprotective mechanism by DEX was not observed in the presence of 5-hydroxydecanoate, a mito-KATP channel blocker, which suggests that its pharmacological target involves mitochondrial components (Yuan et al., 2017).

Acute lung injury (ALI) is a common condition in critical patients, and lipopolysaccharide (LPS) is the most important pathogen that determines the development of ALI in sepsis (Gonzales et al., 2014). Dexmedetomidine has been reported to attenuate LPS-induced ALI in rats (Hanci et al., 2012; Fu et al., 2017). Specifically, DEX (50 µg/l 30 min prior to LPS cell treatment) diminished LPS-induced mitochondrial dysfunction by preventing the reduction in cellular ATP levels and MMP in rat type I alveolar epithelial cells (AECs) (Fu et al., 2017). In addition, the same concentration of DEX markedly reduced the LPS-induced mitochondrial-dependent apoptotic pathway in AECs, as the 2α-adrenoreceptor agonist decreased the cytosolic cytochrome c and caspase 3 activity (Liu et al., 2018). *In vivo* dexmedetomidine (50 ug/kg, 30 min prior LPS administration) also decreased LPS-induced apoptosis, as demonstrated by attenuating DNA fragmentation, activation of caspase 3, Bax upregulation and Bcl-2 downregulation in lungs. Furthermore, DEX markedly diminished LPS-induced oxidative stress, as evidenced by the downregulation of cellular ROS in AECs and lipidperoxidation levels in serum (Fu et al., 2017). Moreover, dexmedetomidine treatment inhibited hyperoxia or the LPS/ATP-induced decrease of MMP and mitochondrial ROS production in RAW264.7 cells; therefore, through this mechanism, dexmedetomidine restrained the NLRP3 inflammasome activation-mediated increased release of IL-1β, IL-18, and TNF-α (Zhang et al., 2017). Dexmedetomidine may contribute to reducing ALI, by preventing the activation of macrophages through a mechanism that involves mitochondrial protections or by inducing neutrophil cell death, also by targeting the mitochondria (Kishikawa et al., 2008). Dexmedetomidine (100 ng/ml, 24 h incubation) has been shown to induce apoptosis in neutrophils through mechanisms involving caspase cascade activation and mitochondrial intrinsic pathway triggered by decreasing the MMP (Kishikawa et al., 2008). In the intrinsic pathway, loss of MMP induces the mitochondrial disruption, and mitochondrial pro-apoptotic proteins are released into the cytosol, triggering the activation of caspase-9 (Wang and Youle, 2009). Interestingly, DEX-induced apoptosis is unlikely to be 2α-adrenoreceptor-mediated, as yohimbine, a 2α-adrenoreceptor antagonist, did not attenuate its pro-apoptotic effects (Kishikawa et al., 2008). As the pro-apoptotic effect of dexmedetomidine is evidenced at a concentration 100-times greater than is considered clinically relevant (1 ng/ml), the clinical administration is safe for ICU patients.

**Figure 1:**
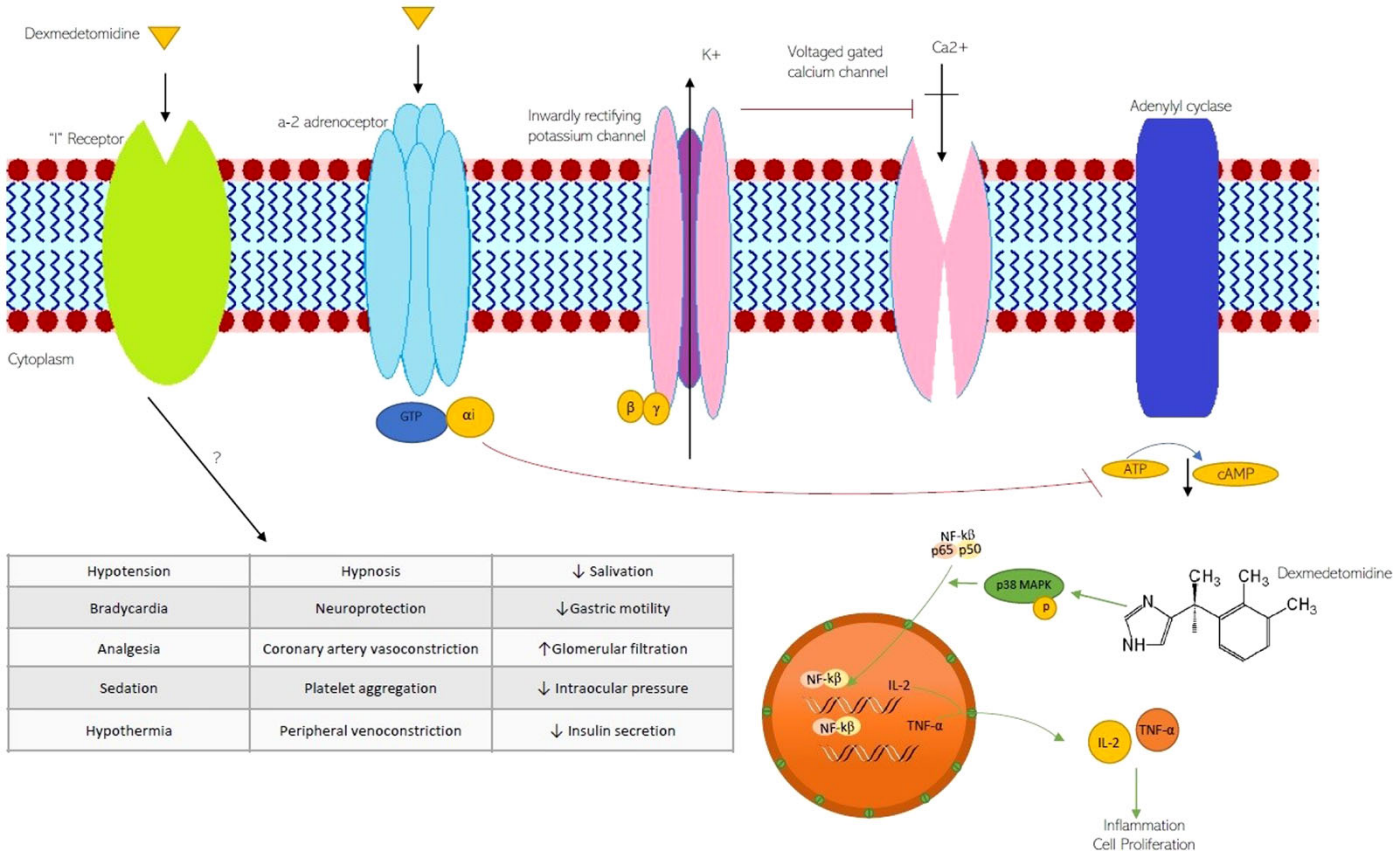
The molecular signaling pathways trigger by DEX and pathophysiological effects on ion channels and G-protein-coupled receptors. K^+^, potassium channel; Ca^2+^ calcium channel; GTP guanosine triphosphate; αi alpha inhibitory unit; cAMP c- adenosine monophosphate; NF-κB nuclear factor kappaB; MAPK, Mitogen-Activated Protein Kinases.

The molecular signaling pathways trigger by DEX, and pathophysiological effects are shown in **Figure 1**.

## Clinical Efficacy for the Use of DEX in Different Clinical Settings

Diverse effects of DEX include sedation, analgesia, anxiolysis, sympatholysis, cardiovascular stabilization and reduction of anesthetic requirements with preservation of respiratory function (Bhana et al., 2000; Kaur and Singh, 2011; Mahmoud and Mason, 2015). Although labeled for short-term sedation only (24 h or less), its use has been demonstrated to be safe for long-term sedation in critically ill patients (Ozaki et al., 2014), in whom pharmacokinetics is often unpredictable (Weerink et al., 2017). Other important benefits are its easy dosage titration (reducing oversedation risk), its several routes of administration, and minimal clinical impact of its metabolites (Devlin, 2008; Gerlach et al., 2009). These features have given DEX an advantage over other sedatives, such as opioids and benzodiazepines. Its clinical use has been focused on two major scenarios: periprocedural sedation in anesthesia and “light to mild” sedation in the ICU (Arcangeli et al., 2009).

### DEX as Peri-Procedural Sedation in Anesthesia

The unique properties of DEX make it suitable for sedation and analgesia during the entire perioperative state in a wide range of procedures (**Table 1**). DEX can be used as premedication, as an anesthetic adjunct for anesthesia or as a postoperative sedative process. This versatility is further enhanced by the property of being compatible with practically all administration routes used in usual perioperative care (**Table 2**). This is particularly useful in pediatrics, non-collaborative patients, or those with insufficient venous access (Scott-Warren and Sebastian, 2016; Weerink et al., 2017; Sottas, 2017).

**Table 1:** Reported use of DEX in several settings of perioperative care.

Epidural anesthesia	Schnaider et al., 2005
Spinal anesthesia	Kanazi et al., 2006
Caudal anesthesia	El-Hennawy et al., 2009
Peripheral nerve block	Dai et al., 2018
Intraarticular use (Arthroscopic surgery)	Al-Metwalli et al., 2008
Intensive care unit sedation	Riker et al., 2009
Transesophageal echocardiography	Cooper et al., 2011
Colonoscopy	Jalowiecki et al., 2005
Awake carotid endarterectomy	Bekker et al., 2004
Awake intubation	Bergese et al., 2007
Vitreoretinal surgery	Ghali et al., 2011
Tonsillectomy/Adenoidectomy	Olutoye et al., 2010/Li et al., 2018
Shockwave lithotripsy	Kaygusuz et al., 2008
Attenuated response to tracheal intubation and extubation	Guler et al., 2005
Post-operative analgesia	Venn et al., 1999
Cardiac surgery	Wijeysundera et al., 2003
Neurosurgery	Bekker et al., 2008/Bekker et al., 2013
Sedation in obese patients	Hofer et al., 2005
Use in MRI	Mason et al., 2008
Endoscopic retrograde cholangio-pancreatography	Lu et al., 2018
Ureteroscopy	Shariffuddin et al., 2018

**Table 2:** Dosage of DEX used in several administration routes.

Route	Dosage	
Intravenous	For ICU sedation 0.2–1.4 mcg/kg/h, may start 1.0 mcg/kg bolus For Procedural sedation 0.2–1 mcg/kh/h	Riker et al., 2009
Intranasal	1–2 mcg/kg	Cimen et al., 2013
Buccal	1–2 mcg/kg	Cimen et al., 2013
Intramuscular	2.5 mcg/kg	Sun et al., 2014
Spinal	0.1–0.2 mcg/kg	Kanazi et al., 2006
Epidural	1–2 mcg/kg	Schnaider et al., 2005
Peripheral nerve block	1 mcg/kg	Obayah et al., 2010

Several meta-analyses have evaluated the use of DEX in the perioperative period. Piao and Wu (2014) focused on cardiovascular stress response to surgical injury. They found patients receiving DEX had significantly lower blood pressure and heart rate. Conversely, they also found more hypotension and bradycardia. In an earlier publication, Sun et al. (2008) found that DEX reduced the amount of intravenous and inhaled anesthetics, but did not reduce muscle relaxant dosages. Other authors have shown a reduction in postoperative delirium (see below), pain (Schnabel et al., 2013a; Schnabel et al., 2013b; Huang et al., 2015) and shivering (Lewis et al., 2015; Liu et al., 2015; Hoffman and Hamner, 2016). The effect of DEX on postoperative shivering is still not well understood (Lopez, 2018). Although some positive results have been shown in clinical trials, the evidence is still regarded as poor. Moreover, DEX is not superior to other anti-shivering agents, such as fentanyl, meperidine, tramadol or clonidine.

### DEX in the ICU

Sedatives are administered to critically ill patients to alleviate discomfort, prevent agitation-related harm, decrease metabolic demand (reducing O_2_ consumption and CO_2_ production), and improve tolerance and adaptation to mechanical ventilation (Taittonen et al., 1997; Chlan et al., 2017). In order to reduce the adverse events associated with sedative drugs, clinical guidelines have been issued (Barr et al., 2013; Devlin et al., 2018). One of the principal indication for the use of DEX is to alleviate the pain and induce sedation in critical patients by targeting light levels of sedation. However, its administration is not recommended in acute respiratory distress syndrome (ARDS), shock or intracranial hypertension. These recommendations match in many aspects, the pharmacological and clinical properties of DEX, explaining its increasing use in intensive care units (DeBiasi et al., 2015).

Among the main reported benefits associated with DEX use in the ICU are shorter ICU stays and mechanical ventilation duration (Chlan et al., 2017), less use of benzodiazepines, and lower incidence of delirium (Su X. et al., 2016; Nunes et al., 2018). Conversely, bradycardia and hypotension occurrence have been described in ICU patients with a minimal impact on mortality (Barr et al., 2013; Constantin et al., 2016; Devlin et al., 2018).

### DEX in Invasive Mechanical Ventilation

As noted earlier, one of the main indications of sedoanalgesia in the ICU is to improve tolerance and adaptation to mechanical ventilation, as the prevalence of anxiety and agitation in ventilated patients of up to 80% has been reported (Tate et al., 2012; Chlan et al., 2017). Conversely, in the case the use of oversedation in ICU patients the deleterious effects are evidenced. In this case, the clinical effects include: increase in the occurrence of delirium, immobility, respiratory muscle atrophy, long-term mechanical ventilation duration, prolonged of ICU stays, and increased costs and even a rise in mortality (Rodrigues Júnior and do Amaral, 2004; Wunsch, 2012; Kress and Hall, 2014; Reade and Finfer, 2014). Thus, the optimal management of sedation in this scenario has been the subject of extensive debate. For a better understanding of this issue, it is essential to distinguish between two different scenarios where mechanical ventilation is frequently used. The first involves an unstable patient, where deep sedation is required, often associated with a neuromuscular blockade, and where protective ventilation is sought. This patient needs complete suppression of spontaneous respiratory effort, with the physiological objective of reducing the associated pulmonary injury (Yang and Kang, 2017), decreasing metabolic demand (Kumba and Van der Linden, 2008) or reducing intracerebral pressure (Oddo et al., 2016). Examples of this situation include ARDS, severe shock, or unstable intracranial hypertension. In these settings, the use of DEX does not seem to have a role.

In the second scenario, the patient has a less severe condition than the one previously described. Clinical stability has been reached, the cause that led to ICU admission is in clear resolution, resuscitation has been completed, or the effort for strict “pulmonary protection” has ceased. In other words, the patient has entered the “dereanimation” phase (Silversides et al., 2017), where the target is the reduction of the morbidity attributed to the interventions carried out in the ICU. In this scenario, it is highly likely that a more superficial sedation, which favors controlled spontaneous ventilatory effort, mobility, and awakening, will produce better clinical outcomes. DEX has a role in this setting and has indeed shown optimal outcomes in clinical trials (Fraser et al., 2013; Hayashida et al., 2016).

In summary, in patients undergoing mechanical ventilation in the ICU who do not require deep sedation, the use of DEX is associated with fewer days in the ICU, shorter time to extubation and a decrease in the incidence of delirium, although no differences in mortality have been shown (Reade and Finfer, 2014; Su X. et al., 2016; Nunes et al., 2018).

### DEX in Non-Invasive Ventilation

Non-invasive ventilation (NIV) has shown to be beneficial in the treatment of acute respiratory failure associated with acute cardiogenic pulmonary edema, chronic obstructive pulmonary disease and postoperative care, amongst others (Jaber et al., 2010; Osadnik et al., 2017; Bello et al., 2018). These benefits are mainly explained by decreasing the need for intubation and connection to mechanical ventilation (Osadnik et al., 2017; Bello et al., 2018). One of the leading causes of NIV failure is agitation, non-tolerance, and non-cooperative patient, reaching rates of up to 22% (Carlucci et al., 2001). In these situations, light sedation has been proposed, and some series have reported its use in up to 20% of patients (Hilbert et al., 2015). DEX has been used in this setting considering its sedative and analgesic effect, without depressing the respiratory center or interfering with airway protection, in addition to its excellent safety profile (Hilbert et al., 2015). Current evidence suggests that it is a safe choice, especially in centers where there is a specialized NIV team (Hilbert et al., 2015). Regarding its effectiveness, the literature is scarce and otherwise contradictory.

### DEX and Delirium

Delirium is a common problem in clinical medicine, which is defined as an acute alteration of attention and awareness, attributable to a general medical state (American Psychiatric Association, 2013). In a comprehensive review (Inouye et al., 2014), the highest incidence rates were observed in ICU and postoperative care settings, ranging between 19 and 82%. Although usually transient, it is associated with worse clinical outcomes, including longer hospital length of stay, increased costs, more days on mechanical ventilation, and long-term cognitive impairment and dependence (Pun and Ely, 2007). Moreover, patients who develop delirium in the ICU experience a threefold increase in six-month mortality risk compared to matched, non-delirious ICU patients (Ely et al., 2004). Among the modifiable risk factors, the use of sedatives and opioid agents has been identified as one of the most relevant, conferring a relative risk of 4.5 in validated predictive models (Inouye et al., 2014). Hence, it is not surprising other drugs have been tested to provide analgesia or light sedation with a lower delirium risk. Several systematic reviews and meta-analyses have evaluated DEX versus opioids, benzodiazepines, and propofol in terms of delirium occurrence (Mo and Zimmermann, 2013; Pasin et al., 2014; Duan et al., 2018; Flükiger et al., 2018). The most recent one by Flükiger et al. (2018) included 25 trials regarding delirium prevention and three regarding its treatment. The authors found that the use of DEX was associated with significantly minor incidence of delirium when compared to the placebo group, standard sedatives, or opioids. In terms of treatment, the small sample and different comparators made it impossible to argue in favor of or against its use. Recently, prophylactic nightly use of DEX has shown to reduce delirium (Skrobik et al., 2018). The results of this industry-funded, two-center, double-blind, placebo-controlled trial have not been replicated but remain promising.

Current published clinical guidelines (Devlin et al., 2018) do not directly support the use of DEX for delirium prevention. Still, they do acknowledge the use of DEX, as a sedoanalgesia, is associated with a lower incidence of delirium compared to strategies based on benzodiazepines or opioids. No recommendations are made concerning its prophylactic nightly use. Finally, regarding delirium treatment, guidelines suggest the use of DEX in patients undergoing mechanical ventilation, in which agitation precludes weaning/extubation.

### DEX in Neurological Injury

Scare evidence exists for the neuroprotective effects of DEX in children following neurological injury (Alam et al., 2017). In addition, there are no trials showing the long-term effects of DEX on memory acquisition in children; this is probably due to the inherent challenges associated with designing trials in children. As a result, clinical evidence for the neuroprotective effects of dexmedetomidine in children is limited to cases of delirium following anesthetic infusion, and sedation in critical care patients (Sottas, 2017).

Intracranial lesions or trauma-related damage activate immune inflammation and neuroendocrine responses, causing ischemic brain injury. Studies have shown that inflammatory pathways mediated by neuroendocrine hormones and proinflammatory cytokines is implicated in the pathophysiology of ischemic brain injury. Alpha2-adrenoceptor agonist, dexmedetomidine, is used as neuroprotectant in anesthetic procedures (Jiang et al., 2017). However, meta-analyses to evaluate the neuroprotection of dexmedetomidine against ischemic brain injury are still lacking.

DEX exerts protective effects on brain injury through multiple mechanisms, such as decreasing local inflammation and improving the cerebral oxygen metabolism (Oh-Nishi et al., 2009). These mechanisms underlay the effect of DEX in reducing the ischemic zona and infarct size in an animal model of brain hypoxia-ischemia injury (Ren et al., 2016). Experimental approaches indicated that DEX decreases tumor necrosis factor (TNF)-α, interleukin (IL)-6 levels; and maintains a high level of superoxide dismutase (SOD) activity (Wang et al., 2015). In addition, DEX achieves hemodynamic stability and attenuates the stress-related increase of intracranial pressure (Schomer et al., 2019). In this case, the modulation of inflammatory and stress hormones levels are all relevant predictors of clinical outcome following ischemic brain injury (Sahay et al., 2018).

### DEX and Sepsis

Other effects of DEX have been shown *in vitro,* such as anti-inflammatory mechanism in sepsis. These effects are mediated by its action on adrenergic receptors that trigger NF-kB inhibition and level reduction of TNF-α, IL-6, IL-8, and HMGB1 (Kawasaki et al., 2013). Moreover, *in vivo* sepsis, the vagus nerve and α7nAChR-mediated cholinergic anti-inflammatory pathway are required for the anti-inflammatory effect of DEX (Xiang et al., 2014).

Patients that develop sepsis may have marked respiratory and cardiovascular depression. Early stages of sepsis are associated with a drop in the systemic vascular resistance, high cardiac output, and a hypercontractile state (Yuki and Murakami, 2015). The improvement of the sepsis management often requires that patients receive anesthesia as well as analgesia. This is due to the unstable cardiovascular state, changes that exacerbate the cardiac dysfunction. Anesthetic drugs in this setting, should not inhibit compensatory hemodynamic responses. Unfortunately, current intravenous drugs and inhalational pharmacological agents do not meet these considerations (Yuki and Murakami, 2015; Cata et al., 2019). However, DEX seems promising in this context, and mechanistic, pharmacodynamic, and pharmacokinetic studies with DEX in critical septic patients have increased in recent years. Recent evidence in septic shock sedated patients have shown that for a comparable level of sedation, switching from propofol to dexmedetomidine resulted in a reduction of norepinephrine requirements in septic shock patients (Morelli et al., 2019).

**Table 3** shows the benefits of adding DEX in different clinical settings.

**Table 3:** Benefits of adding DEX in different clinical settings.

Clinical scenario	Benefits of using DEX	References
Peri-procedural sedation	Effective sedation in diverse procedural scenarios. Diverse administration routes. Reduces sympathetic and stress response to surgery. Sparing effects of anesthetic agents Reduces postoperative delirium, shivering and pain	Dyck et al., 1993; Khan et al., 1999; Bhana et al., 2000; Dutta et al., 2000; Venn et al., 2002; Anttila et al., 2003; Petroz et al., 2006; Sun et al., 2008; Potts et al., 2009; Su et al., 2010; Lin et al., 2011; Iirola et al., 2011b; Schnabel et al., 2013a; Schnabel et al., 2013b; Piao and Wu, 2014; Cortinez et al., 2015; Hannivoort et al., 2015; Lewis et al., 2015; Liu et al., 2015; Mahmoud and Mason, 2015; Yoo et al., 2015; Zhang et al., 2015; Hoffman and Hamner, 2016; Scott-Warren and Sebastian, 2016; Su X. et al., 2016; Wiczling et al., 2016; Liu et al., 2017; Sottas, 2017; Weerink et al., 2017
ICU	Reduces ICU stays and duration of mechanical ventilation Sparing effects of benzodiazepines Delirium prevention	Bhana et al., 2000; Su X. et al., 2016; Chlan et al., 2017; Nunes et al., 2018; Flükiger et al., 2018
Invasive Mechanical Ventilation	Shorter Time to extubation and ICU stay. Decrease incidence of delirium	Fraser et al., 2013; Reade and Finfer, 2014; Su et al., 2016; Hayashida et al., 2016; Nunes et al., 2018
Non-invasive Ventilation	Sedation and analgesia without respiratory depression	Hilbert et al., 2015
Delirium	Reduces incidence of delirium in ICU and postoperative period	Mo and Zimmermann, 2013; Pasin et al., 2014; Mahmoud and Mason, 2015; Duan et al., 2018; Flükiger et al., 2018; Skrobik et al., 2018
Neurological Injury	Potential neuroprotection in young children. Potential neuroprotection in Ischemic brain injury	Ren et al., 2016; Alam et al., 2017; Sottas, 2017; Schomer et al., 2019
Sepsis	Anti-inflamatory effects in sepsis. Potential hemodynamic stability	Xiang et al., 2014; Morelli et al., 2019

## Concluding Remarks

Dexmedetomidine is a highly selective alpha-2 receptor agonist. Since its approval by the FDA for sedation in the ICU, its applications have expanded significantly because of its unique mechanism of action, minimal effect on the respiratory drive, and ideal safety profile. Its applications include premedication for pediatric and adult patient populations, procedural sedation and monitored anesthesia care, adjuvant for general and regional anesthesia, perioperative pain control, and postoperative delirium. Clinical evidence show that dexmedetomidine has little effect on the respiratory drive; it still can cause airway obstruction in combination with other anesthetic agents (Nguyen et al., 2017).

Preclinical studies with DEX on murine models have revealed protective effects, including the inhibition of proinflammatory cytokine production (Taniguchi et al., 2004). In *vitro* studies with human whole blood samples have shown that DEX significantly suppresses Lipopolysaccharide induced proinflammatory mediators dose-dependently (Kawasaki et al., 2013). Clinical investigations with human subjects evaluating the effects of DEX on serum inflammatory cytokines during perioperative conditions show inconsistencies in the outcomes (Liu and Qian, 2013; Liang et al., 2017)

Multiple trials and meta-analyses also suggest that DEX use is associated with less delirium and cognitive disturbance, faster weaning from mechanical ventilation, and decreased time to extubation compared with other sedative agents (Shehabi et al., 2019; Yamamoto et al., 2019). As such, DEX seems to be an ideal primary anesthetic agent in the sedation of intubated and mechanically ventilated critical patients.

Regarding their molecular effects, DEX pre-, intra- and postconditioning treatments showed neuroprotective and cardioprotective effects reduced cell necrosis, although only preconditioning showed antiapoptotic mechanisms (Ren et al., 2016; Deng et al., 2019). Dexmedetomidine treatments also reduced IL-6 and TNF-α levels, especially in the preconditioning groups. Oxidative stress is attenuated with all DEX-preconditioning treatments, but only with the higher dose in the intraconditioning group, and no effects were observed in the postconditioning (Rodriguez-Gonzalez, 2016; Cheng et al., 2018). According to these changes, conditioning strategies increased BDNF levels and attenuate mitochondrial dysfunction *in vivo* and *in vitro* models of IR injury (Huang and Jiang, 2019).

**Figure 2:**
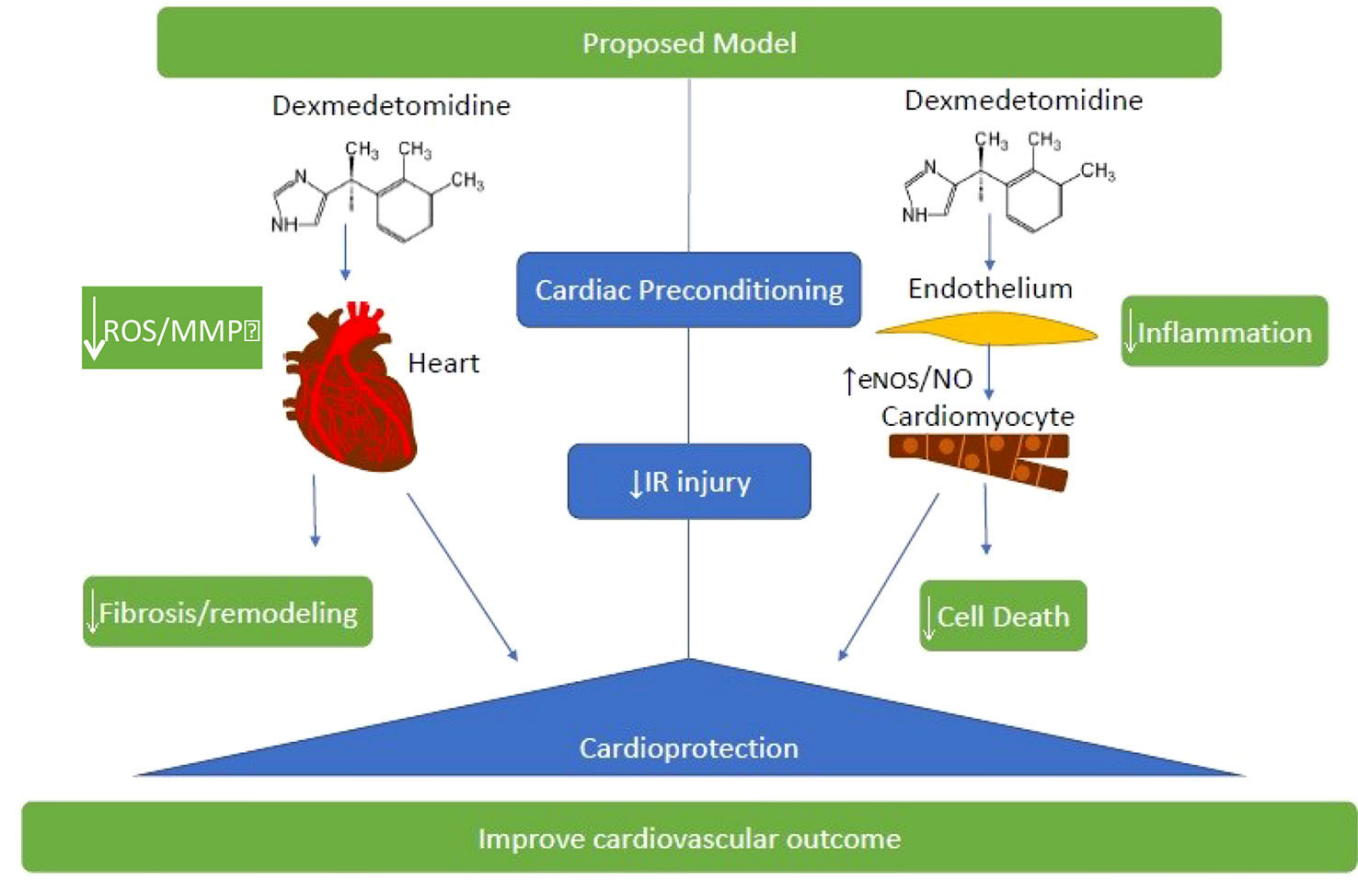
Cardiac preconditioning effects associated with DEX administration validated in animal models. These strategies that determine an attenuation of oxidative stress and inflammation in cardiac tissue are very important to improve the clinical outcome of patients subjected to ischemia-reperfusion (IR) injury. ROS, reactive oxygen species; MMP Matrix metalloproteinases; eNOS, endothelial nitric oxide synthase; NO, nitric oxide.

Cardiac preconditioning effects associated with DEX administration validated in animal models are shown in **Figure 2**.

Overall, evidence shows that DEX may attenuate the mortality and inhibit inflammatory processes, as it enhances the activity of the immune system while reducing its systemic reaction and lowering cytokine levels. Moreover, DEX succeeds in alleviating heart injury during sepsis, acting beneficially for microcirculation, and shows a neuroprotective role by inhibiting cell death pathways. Various molecular signaling pathways, such as mitochondria or the modulation of transcriptional factors, are under study in both *in vivo* and *in vitro* models.

Therefore, we can say with the reviewed evidence that DEX can be used as a drug for the study of translational models from the cellular and animal point of view and in clinical trials with humans which including cardiovascular and ventilator outcomes. The biological activities associated with DEX continues keep growing, and its diverse effects suggest that it may offer a novel therapeutic approach for the treatment of human diseases with I/R involvement.

## Author Contributions

RC: designed the manuscript and the structure of the themes and wrote about animal models and basic targets of DEX. MI and IC: wrote about human pharmacokinetics of DEX. CC-P: provided evidence of DEX on the mitochondrial protection and clinical outcomes. RC: wrote about DEX on cardioprotection. PV-E, RB, and DR: wrote about DEX in different clinical settings. DT and AM: contributed to the design of the figures and the configuration of the references. JGF: contributed to the design of the Tables, figures and the drafting of the conclusions. 

## Funding

This work was supported by “Fondo Nacional de Desarrollo Científico y Tecnológico” (FONDECYT) Grants 1180387, Gobierno de Chile (JGF). Additional funding was provided by Grant SANTANDER-UNIVERSIA 2015, Banco Santander, Chile (RC).

## Conflict of Interest

The authors declare that the research was conducted in the absence of any commercial or financial relationships that could be construed as a potential conflict of interest.
